# Antiphospholipid Syndrome Associated with Nonradiographic Axial Spondyloarthritis

**DOI:** 10.1155/2021/4359488

**Published:** 2021-12-01

**Authors:** Jozélio Freire De Carvalho, Antoniella Fernanda Mendanha Sousa

**Affiliations:** ^1^Institute for Health Sciences from Federal University of Bahia, Salvador, Bahia, Brazil; ^2^INPOS, Goiania-GO, Brazil

## Abstract

Herein, we describe a patient with antiphospholipid syndrome (APS) associated with nonradiographic axial spondyloarthritis (NRAS). A 31-year-old woman with a past medical history of uveitis experienced a pulmonary thromboembolism in March 2014 and was treated with rivaroxaban (20 mg/day). Five months later, she started complaining of low back pain. The results on contrast-enhanced sacroiliac magnetic resonance imaging were normal. Laboratory tests revealed positive HLA-B27 and the presence of lupus anticoagulant and IgM anticardiolipin. The diagnoses of APS and NRAS were made. The patient was treated with rivaroxaban for APS and sulfasalazine (2 g/day), respectively. As she showed the presence of lupus anticoagulant antibodies in blood, she did not receive nonsteroidal anti-inflammatory drugs. After 6 months, the patient was asymptomatic, without lumbar pain; she also showed normalization of the erythrocyte sedimentation rate and the C-reactive protein and vitamin D levels, good control of lumbar pain, and no new uveitis episodes. The APS was also stable. To the best of our knowledge, this is the first reported case of NRAS associated with APS.

## 1. Introduction

Antiphospholipid syndrome (APS) is a systemic autoimmune disease characterized by recurrent thrombosis associated with obstetric complications and/or thrombocytopenia in the presence of persistent antiphospholipid antibodies [1]. This coagulopathy, when isolated, is called primary APS, or it may be associated with other autoimmune conditions (i.e., secondary APS), commonly with systemic lupus erythematosus [1].

In an extensive study with 1,000 patients with APS, the diseases associated with APS, apart from primary APS in 53.1% of the cases, were lupus in 36.2% of the cases, lupus-like syndrome in 5%, Sjögren syndrome in 2.2%, rheumatoid arthritis in 1.8%, systemic sclerosis in 0.7%, systemic vasculitis in 0.7%, and dermatomyositis in 0.7% [2].

APS with spondyloarthritis is quite rare; only five cases have been previously reported [3–7]. To the best of our knowledge, no case of nonradiographic axial spondyloarthritis (NRAS) associated with APS has been reported. Therefore, herein, we report the first case of APS associated with NRAS.

## 2. Case Report

A 31-year-old woman had a past medical history of obesity, dyslipidemia, and breast nodules, as well as a family history for vitiligo in her sister, multiple sclerosis in her cousin, and pulmonary thromboembolism in an aunt who died of it. She also had a childhood history of transient polyarthralgia and had received glucocorticoids. When she was 23 years of age, she was diagnosed with uveitis in her left eye. In March 2014, she started complaining of radiating pain in her left flank and left shoulder while breathing, and a diagnosis of pulmonary thromboembolism was confirmed on the basis of the results of perfusion scintigraphy and pulmonary angiotomography. The patient was hospitalized and treated in an intensive care unit, receiving intravenous heparin. She was discharged after being prescribed rivaroxaban (20 mg/day). In September 2014, the patient started complaining of inflammatory low back pain that had persisted for more than 3 months. Physical examination demonstrated a blood pressure of 140/80 mmHg, weight of 95.15 kg, height of 1.64 m, and body mass index of 35.38 kg/m^2^. The sacroiliac joints were normal on the physical examination. The results on contrast-enhanced sacroiliac magnetic resonance imaging were normal. Laboratory tests revealed the presence of HLA-B27, as well as positive results for lupus anticoagulant antibodies (in February and June 2014) and low titer IgM anticardiolipin antibodies (15.7 MPL; positive value > 10 MPL); the results for IgG anticardiolipin antibodies were negative. The patient was heterozygous for the presence of both mutant prothrombin and methylenetetrahydrofolate reductase. All other thrombophilia factors were within the normal ranges, including antithrombin III (128%), protein C (170%), protein S (130%), and homocysteine (9.4 *μ*mol/L); factor V Leiden was absent. The patient was negative for antinuclear antibodies, anti-Sm, anti-dsDNA, anti-RNP, anti-Ro/SS-A, anti-La/SS-B, anti-CCP, rheumatoid factor, and ANCA; complement levels were within the normal ranges. The 25-OH-vitamin D level was 20.4 ng/mL (normal >30 ng/mL), the erythrocyte sedimentation rate (ESR) was 30 mm/h, and the C-reactive protein (CRP) level was 30.4 mg/L. A diagnosis of APS was made on the basis of the occurrence of pulmonary thromboembolism and the results of at least two tests that showed positive lupus anticoagulant antibodies over 12 weeks, and a diagnosis of NRAS was made on the basis of the observation of inflammatory low back pain for more than 3 months in a patient aged <45 years, as well as the positive result for HLA-B27, the presence of uveitis, and high levels of CRP. The patient was treated with rivaroxaban for APS and sulfasalazine (2 g/day); a single subcutaneous injection of vitamin D3 (600,000 IU) was also added as part of her treatment. As the patient showed the presence of lupus anticoagulants in blood, she did not receive nonsteroidal anti-inflammatory drugs. After 6 months, she was asymptomatic, with good control of lumbar pain, as well as normalized ESR and CRP and vitamin D levels; she also did not show any new episodes of uveitis. The APS was also stable without any new clinical or laboratory features.

## 3. Discussion

To the best of our knowledge, herein, we describe the first patient with APS and NRAS.

Antiphospholipid antibodies have been observed in patients with spondyloarthritis. In a study of 84 patients with axial spondyloarthritis and 40 healthy controls, antiphospholipid antibodies were more frequently observed in patients with axial spondyloarthritis (29% vs. 5% in the controls, *p* < 0.002). Importantly, no patient had thrombosis or other clinical manifestations of APS [8]. However, antiphospholipid antibodies have not been detected in patients with axial spondyloarthritis in other studies [9].

NRAS is more frequently observed in women than in men. Although patients with NRAS have a shorter disease duration and do not show radiological alterations, they experience a substantial illness burden, with self-reported disease activity and functional impairments comparable to those of patients with radiographic disease. Interestingly, radiographic progression was observed in approximately 10% of these patients over 2 years [10].

The first description of APS in a patient with axial spondyloarthritis was reported in 1991 by Blanchard et al. in a 60-year-old man with leg ulcers and persistent lupus anticoagulants [5]. Two other cases of APS associated with axial spondyloarthritis have been described in the medical literature by Mateo et al. in 1992 [3, 4]; the first patient with axial spondyloarthritis developed a pons infarction associated with high titers of anticardiolipin antibodies, and the second patient had deep vein thrombosis with the presence of lupus anticoagulant antibodies [3, 4]. The fourth case of APS in a patient with axial spondyloarthritis was described in 1993; the patient had axial spondyloarthritis with deep venous thrombosis and positive lupus anticoagulant antibodies [6]. The fifth case of APS in a patient with axial spondyloarthritis was reported in a 67-year-old woman with spondyloarthritis who was treated with adalimumab. After the 3rd dose of this biological drug, she experienced ischemic changes in her distal fingers and toes, showed positivity for IgM anticardiolipin antibodies, and showed the appearance of antinuclear antibodies and ANCA anti-dsDNA. The authors linked the autoimmunity and thrombosis to a drug-induced manifestation secondary to adalimumab use [7].

In conclusion, herein, we describe a rare case of APS with NRAS. The patient was treated with anticoagulants and sulfasalazine and had good outcomes for both diseases.

## Additional Points

*Highlights*. Antiphospholipid syndrome (APS) is characterized by recurrent thrombosis and/or obstetric events.Nonradiographic axial spondyloarthritis (NRAS) is a type of spondyloarthritis characterized by the clinical features of spondyloarthritis but no imaging evidence of sacroiliitis or spine involvement. No case of APS associated with NRAS has been previously described.We describe the first case of APS with NRAS.
